# Lack of CD117 and rare bcl-2 expression in stomach cancer by immunohistochemistry. An immunohistochemical study with review of the literature

**DOI:** 10.1186/1746-1596-1-7

**Published:** 2006-05-16

**Authors:** Masoud Mireskandari, Ali Fakhr Shafaii, Gian Kayser, Klaus Kayser

**Affiliations:** 1 Afzalipour Hospital, Pathology Department, Kerman University of Medical Sciences, Kerman, Iran; 2 Department of Pathology, University Freiburg, Freiburg, Germany; 3 UICC-TPCC, Charite, Berlin, Germany

## Abstract

**Background:**

Gastric adenocarcinoma is one of the most frequent malignancies worldwide including Iran. This study was designed to immunohistochemically evaluate the CD117 and bcl-2 expression in gastric carcinomas and their potential use as therapeutic targets in the treatment of patients with advanced stage gastric cancer.

**Materials and methods:**

Representative paraffin blocks obtained from 38 operated gastric adenocarcinoma patients were retrieved from Afzalipour Hospital pathology department archive, Kerman, Iran. Immunohistochemical analysis (IHC) for CD117 was carried out in all cases including negative (normal gastric epithelium) and positive (Gastrointestinal Stromal Tumor) controls. In addition, the cases were evaluated immunohistochemically for apoptosis-related protein (bcl-2), to evaluating a potential association of CD117 expression with the cell proliferation regulatory pathways.

**Results:**

No positive reaction for CD117 was seen in gastric carcinoma tumor cells irrespective to the cell type, grade, and stage, proliferation and apoptosis rate. Expression of bcl-2 was observed in only one case.

**Conclusion:**

We conclude that CD117 overexpression detectable by immunohistochemistry does not play a significant role in gastric carcinoma pathways and development, although overexpression at the gene level and/or mutated CD117 expression might exist. Thus, it is unlikely that the CD117 pathway is of clinical significance in gastric carcinoma patients.

## Background

Gastric carcinoma is the second most frequent malignancy in the world constituting about 10% of newly diagnosed malignancies and accounting for more than 12% of cancer deaths [8]. It is the disease of the elderly with a median age of 72 years in American patients at the time of diagnosis [8]. Immunohistochemically (IHC), almost all tumors are positive for cytokeratin and epithelial membrane antigen [43]. Furthermore, gastric carcinomas have been shown to be positive for following factors in different proportions: Pancreatic Secretory Trypsin Inhibitor (76% and 89% in intestinal and diffuse types respectively) [19], carcinoembryonic antigen (30–55 %), Villin (47 %), Beta Catenin (63%), CD44 (55–58%), [22,43] and Placental Alkaline Phosphatase (23%) [49]. Mutations of the p53 protein and abnormalities of the Adenomatous Polyposis Coli (APC) gene were found in about 50% of cases [1]. In the literature, only one study examining the expression of CD117 in gastric carcinomas exists to our knowledge until now [18]. This study reports an expression of the protooncogene c-kit and its ligand stem cell factor (SCF) in gastric carcinoma cell lines [18]. In vivo, the potential expression of the protein in human gastric carcinoma has not been analyzed, and no statement about the potential clinical significance of the in vitro study has been given to our knowledge.

Classically gastric carcinomas are classified to intestinal and diffuse type according to the Lauren classification [29]. Although the role of adjuvant therapies in advanced gastric cancer is under debate, generally surgical resection is considered as the standard therapy and response to other methods of treatment is poor [46].

Gastric carcinomas are usually detected at advanced tumor stages. The factors involving the miss an early diagnosis comprise anatomical and clinical reasons. Anatomically, the stomach has a very large luminal space and the tumors can reach quite a large size prior to inducing pain or symptoms of obstruction. From the clinical point of view, symptoms of gastric carcinoma are equivalent to benign lesions such as chronic gastritis or peptic ulcer. Often, these lately diagnosed tumors are no longer amenable to surgery. Population based gastric cancer screening programs have not been implemented in most countries due to lack of sensitive and specific serum tumor markers, high economic burden, and poor compliance of routine gastroscopy.

Thus, it seems reasonable to investigate in new therapeutic modalities for advanced gastric cancer. Immunotargeting and blocking specific tumor growth pathways is one of the new promising strategies. Successful examples include regimes against CD20 and Her-2-neu in non-Hodgkin's B cell lymphoma (NHL) and advanced stage breast carcinoma, respectively [5,23]. Chimeric anti-CD20 antibody (rituximab) sensitizes B cells in NHL to inducing apoptosis by cytotoxic substances, for example, by diminishing the Bcl-x expression [23]. Inhibitor of Her-2-neu protein (trastuzumab) has been reported to induce a 19% response rate in metastatic breast carcinoma patients [5]. An additional agent is associated with the expression and inhibition of CD117 (c-kit), and has been effectively used in various neoplasms such as hematological malignancies and gastrointestinal stromal tumors (GIST). Thus, these inhibitors could be potentially considered as a new target for therapeutic regimes in gastric carcinomas; however, only if a significant CD117 expression could be demonstrated, i.e., by use of IHC.

### Expression of CD117, c-kit and the stem cell factor (SCF) receptor

CD117 is coded by the c-kit protooncogene on chromosome 4q11-q12 [42]. Being a cell surface protein it is categorized into the third variety of cell receptors named *receptors with intrinsic kinas activity *[10]. It possesses three domains of extra cellular, intracellular, and transmembrane location. Related receptors include the granulocyte colony stimulating factor (G-CSFR) and platelet derived growth factor (PDGFR). The extra cellular domain possesses five immunoglobulin-like areas. The first three are responsible for binding with stem cell factor (SCF). Following a complex formation with SCF the intracellular domain is phosphorylated and new sites are provided for cytoplasmatic messengers e.g. IP3, Src, and ras. Following these events, the cell is committed for entering the S phase and cell proliferation cycle [30].

The expression of the CD117 protein has been identified in different cells types, such as osteoclasts, Langerhans cells, cord blood cell, renal tubules epithelial cells, sweat gland cells, or megakaryocytes [3]. It probably plays a physiologic role in the proliferation of these cells. A significant influence of C-kit in the formation and growth of extremities at embryonic stages has been suspected by animal model studies [41]. Mast cells are dependent on the autocrine circle of CD117 [1], and the loss of CD117 in germinal cells of embryonic testis results in an increased apoptosis of germ cells and infertility in the following life periods [13]. Soluble c-kit can mobilize bone marrow cells into the peripheral blood via the interaction with the vascular cell adhesion molecule 2 (VCAM-2) [35]. Both, SCF and CD117 take influence on the growth of endothelial cells and smooth muscle cells of the umbilical vein [33]. However, the main function of CD117 is the activation of the cell proliferation. Expression of CD117 has been noted in a subset of angiosarcoma cases, a phenomenon that has been compared to the action of an oncofetal protein or its anti-apoptotic effect [9].

In man, CD117 proto-oncogene mutations result in an autosomal dominant disease named piebaldism [24]. Somatic mutations have been recorded in various tumor cell types. The mutation of the c-kit protooncogene has been frequently observed in germ cell tumors, particularly in seminoma [25].

Although an over-expression of CD117 mRNA was noted in cell lines of malignant fibrous histiocytomas [36], IHC evaluation of such tumors for c-kit revealed only one positive reaction out of 43 cases [51]. Immunohistochemically, expression of CD117 also has been demonstrated in leiomyosarcomas of the uterus [39], and has been identified in about 50% of small cell or large cell neuroendocrine lung carcinomas [2]. Over-expression of CD117 is almost always seen in GIST [42]. Immunohistochemically, its expression usually serves for diagnostic differentiation of these tumors from morphologically similar neoplasms [15]. Singular CD117 expression or in combination with that of SCF is noted in several hematopoetic tumors including mast cell leukemia, acute myelogenous leukemia, and other myeloproliferative disorders [10]. In fact, the c-kit expression in acute leukemia can be considered as an indicator of myeloid origin of tumor cells [10] as it is rarely expressed in lymphoid blast cells. Meanwhile its expression in myeloid cells forms the basis for detecting a minimal residual disease (MRD) by flow cytometry [10]. Most tumor cells in Hodgkin's disease including mononuclear and Reed-Sternberg cells express c-kit [10]. Colon carcinomas rarely display a positive cytoplasmatic reaction for CD117 by IHC [40]. C-kit expression varies to a great extent in soft tissue sarcomas, and ranges from 10% in myxoid sarcomas to 60% in melanocytic schwannomas [21]. In human gliomas c-kit is widely expressed and an autocrine pathway of tumor activation and progression by c-kit and its ligand has been suggested herein [17]. Interestingly, normal breast epithelial cells abundantly express c-kit, whereas a loss of c-kit expression has been reported in 80–90% of breast cancer cases [37]. In vitro, the transfer of the c-kit gene into a breast cancer cell line resulted in the suppression of cellular growth [37]. These observations suggest an inhibitory effect of c-kit and its ligand in breast cancer proliferation. One study reports a co expression of c-kit and stem cell factor genes in breast carcinoma and a possible autocrine activation of these cancer cells [20]. A survey of malignant tumors with positive CD117 immunohistochemical reactivity is listed in table 1 according to [22].

**Table 1:** Immunohistochemical detection of CD117 in malignant tumors

**Tumor Type**	**Reactivity (%)**	**Number of cases studied**
Endometrial carcinoma	100	8
Follicular carcinoma of thyroid	100	11
Papillary carcinoma of thyroid	100	9
Merkel cell carcinoma	96	22
Seminoma	96	65
Ovarian serous cystadenocarcinoma	94	16
Renal cell carcinoma, chromophobe	90	74
Osteogenic sarcoma	84	18

### Inhibitors of c-kit

Some endogenous cytokines have shown to posses inhibitory effects on the c-kit receptor [6,28]. Such endogenous inhibitors including TGFβ1 and IL4 were successfully applied in experimental animals and in colon carcinoma cell lines [6,28]. One of the protein tyrosine kinase inhibitors, SU5614 can induce cellular growth arrest and apoptosis in AML by inhibiting the c-kit receptor [45]. STI571 or Imatinib Mesylate is another molecule representing the exogenous inhibitor. Originally applied for the treatment of CML by inhibiting 9;22 the translocation induced tyrosine kinase [44], it later served for an effective treatment of a variety of malignancies including metastasizing gastrointestinal stromal tumors [12]. The treatment with c-kit inhibitors in 21 patients with AML who were partly resistant to chemotherapy was promising too [27]. In vitro studies of small cell lung carcinoma cell lines showed a synergistic effect for c-kit and IGF-1R inhibitors in the induction of apoptosis [9,48]. Other in vitro studies displayed an inhibition of cell proliferation and induction of apoptosis in colon carcinoma [4], breast carcinoma [37], Ewing sarcoma [16] and neuroblastoma cell lines [7]. In addition, a successfully treated case suffering from a hepatocellular carcinoma and liver cirrhosis has been described [38].

### Bcl-2 protein

Bcl-2 protein is a 26 Kd protein with the major role in controlling apoptosis. Bcl-2 production is controlled by a gene located on chromosome 18 [14]. Bcl-2 belongs to a family of proteins which includes Bax protein [31]. Bax counteracts the effects of bcl-2 by increasing cell susceptibility to apoptotic stimuli [31]. In fact it is appeared that the ratio of bcl-2 to Bax proteins control the sensitivity or resistance of many cell types to apoptotic stimuli [31]. Although the role of bcl-2 protein is well known in some forms of low grade lymphomas, including follicular lymphomas [14], its role in other cancers is under investigation. Studies could show the reactivity of tumor cells in gastric carcinoma for bcl-2 with various frequencies [14,50]. A higher frequency of apoptosis was shown in gastric dysplasia rather than in coexisting gastric carcinoma [50]. This observation is suggestive for a specific role of Bcl-2 in gastric carcinoma development through increasing cell life and imposing it to more genetic alterations [50].

Although these observations are now far from practical implementation in gastric cancer treatment, some reports of successful application of apoptosis induction in gastric cell lines are present [32,26].

## Materials and methods

Paraffin blocks of 38 cases with gastric carcinoma operated on between 11^th ^March 1999 and 5^th ^September 2003 were collected from the archive of the Pathology Laboratory of the Afzalipour Hospital. Representative tumor blocks were selected for immunohistochemistry. A paraffin block of a case with a gastrointestinal stoma tumor (GIST) was used for positive control. Immunohistochemistry was performed by application of the Avidin-Biotin Complex (ABC) method for CD117 and bcl-2. Both primary antibodies and LSAB visualization kit were commercially available from DAKO, Copenhagen, Denmark. The heat – induced method (HIER) was applied for antigen retrieval by tris-EDTA buffer PH9. The primary antibody dilution was set to 1/400 for CD117 and to 1/40 for bcl-2 respectively. The deparaffinized and rehydrated slides were incubated with the primary antibodies for 20 minutes, after thorough washing with the secondary antibody for 10 minutes, with streptavidin for 10 minutes, and liquid Diaminobenzydine (DAB) for 5 minutes. Cases were grouped positive if a dark brown reaction in the tumor cells was noted.

## Results

**Figure 1:**
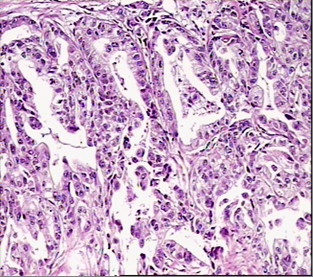
H&E appearance of intestinal type gastric cancer.

**Figure 2:**
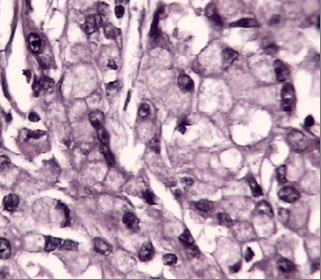
H&E appearance of diffuse type gastric cancer.

The complete list of the 38 cases is given in table 2. The spectrum of the patients' age ranges from 23 to 88 years (mean 56). The sex ratio (men/women) accounts to 1.7:1. Twenty patients underwent a total gastrectomy, the others were treated by subtotal gastrectomy. Six of the tumors developed from the cardiac region (15%), additional six tumors from the corpus area (15%), and 18 cases were located in the antrum (47%). In 6 cases (15%) the tumor extensively infiltrated the stomach, and a detailed location of the tumors could not be identified. One case originated from gastro-esophageal junction and another single case infiltrated both the body and the cardia. According to the Lauren classification 20 cases (52.6%) were grouped into the intestinal type (figure 1) and 17 cases (44.7%) were grouped into the diffuse type (figure 2). One mucinous type was seen (2.7%). Within the group of intestinal type tumors 8 cases were well, and 12 cases moderately or poorly differentiated. Most of the patients were operated on in advanced tumor stages showing only two cases at stage I (5.3%) and 3 cases at stage II (7.9%). The advanced stages include 16 cases (42.1%) at stage IIIA, 15 cases (39.4) at stage IIIB, and 2 cases (5.3%) in stage IV according to the rules of the AJCC/UICC [43].

**Table 2:** Details of demographic, microscopic, pathologic and immunohistochemical findings in the study cases

Ser. No.	Sex	Age	Year of Operation	Location	Cell Type	Grade	Stage
1	F	50	1999	A	D	-	IIIB
2	F	48	1999	DI	D	-	IV
3	M	70	1999	B	D	-	IIIA
4	F	65	1999	A	I	PD	IIIA
5	M	58	1999	A	D	-	IIIA
6	M	59	1999	DI	D	-	IIIA
7	M	75	1999	B	I	MD	IIIA
8	M	28	2000	A	D	-	IIIB
9	M	65	2000	A	D	-	II
10	F	65	2000	A	I	WD	IIIB
11	F	54	2001	A	D	-	IIIA
12	M	70	2001	B	I	PD	IIIA
13	M	70	2001	C	D		IV
14	M	45	2001	C, B	I	PD	IIIB
15	M	60	2001	A	I	MD	II
16	M	51	2001	A	I	MD	IIIB
17	F	67	2001	B	D	-	IIIB
18	F	67	2002	C	D	-	IIIB
19	M	39	2002	C	I	WD	IIIA
20	F	79	2002	B	I	MD	IIIA
21	F	44	2002	DI	D	-	IIIA
22	M	63	2002	A	I	WD	IB
23	M	48	2002	A	I	PD	IIIB
24	M	52	2002	DI	D	-	IIIB
25	M	68	2002	A	I	WD	II
26	M	70	2002	A	I	WD	IIIA
27	M	88	2002	A	I	PD	IIIA
28	F	30	2003	C	I	MD	IIIA
29	M	53	2003	C	I	WD	IB
30	F	53	2003	C	I	MD	IIIB
31	F	60	2003	-	I	PD	IIIB
32	F	48	2003	A	D		IIIB
33	M	42	2003	A	Mucinous	WD	IIIA
34	M	65	2003	B	D	-	IIIB
35	M	45	2003	DI	D	-	IIIA
36	M	23	2003	GEJ	I	WD	IIIA
37	M	58	2003	A	D	-	IIIB
38	F	35	2003	A	I	PD	IIIB

**Abbreviations**: M: male; F: female; A: antral; B: body; C: cardia; DI: diffuse infiltrative; GEJ: gastro-esophageal junction; D: diffuse type; I: intestinal type; PD: poorly differentiated; MD: moderately differentiated; WD: well differentiated.

**Figure 3:**
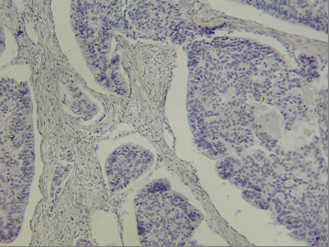
Negative immunohistochemical reaction for CD117 in gastric carcinoma tumor cells.

**Figure 4:**
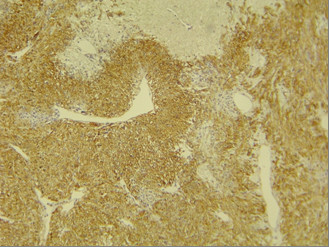
Immunohistochemical staining for CD117 shows strong diffuse positive reaction in control slide (GIST). Note absence of background staining in non-neoplastic elements at the top of image.

**Figure 5:**
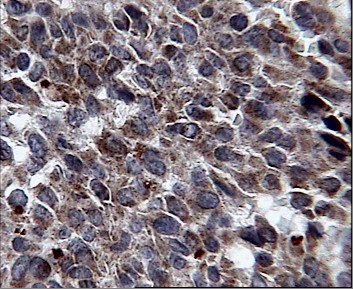
Strong and diffuse cytoplasmic reactivity for bcl-2 in case 11.

The CD117 IHC reaction was negative in all cases including the normal surface and glandular epithelium, dysplastic epithelial cells around the tumor, and the invasive components of gastric carcinoma cells (figure 3) while positive control (GIST) revealed strong and diffuse cytoplasmic reactivity (figure 4). The IHC staining for bcl-2 was also negative in all cases except for one case of a diffuse gastric carcinoma (case no. 11) that showed a distributed strong cytoplasmatic reactivity (figure 5). In 5 cases a scattered and weak cytoplasmatic reactivity was noted for bcl-2, which was considered to be negative too.

## Discussion

The mean age of gastric carcinoma cases in this study (56 years) is significantly lower than that of population based incidence (70 years). Most of our cases had been operated on in advanced tumor stages, and account to a percentage of 86% in stage three and higher.

Obviously, a potential curative surgical and chemotherapeutic treatment of such advanced, poorly differentiated, and aggressive tumors is not possible. Therefore, new therapeutic approaches should be searched for.

One potential regime could be the treatment with imantinib, a substance that originally served for the inhibition of the abl-bcr fusion gene product in chronic myelogenous leukemia. It can also act as a kit tyrosine kinase inhibitor, and, therefore, its expression has been analyzed in GIST and a broad variety of other malignancies [34]. In fact, some oncologists request kit IHC stains when they are confronted with mesenchymal tumors in order to potentially apply imatinib for treatment [34]. Surprisingly, the CD117 expression in gastric carcinomas was not clearly evident although it has been investigated in numerous tumors, such as germ cell or melanocytic malignancies. We could only evaluate one article that describes an over expression of the CD117 gene in gastric carcinoma cell cultures [18].

In our study, none of the studied 38 cases displayed a CD117 expression immunohistochemically. Therefore, it seems to be unlikely, that CD117 plays a remarkable role in gastric carcinoma formation and progression. In addition, its potential application as a new target for therapeutic strategies is questionable. This statement holds true, even if more sensitive methods like fluorescent in situ hybridization (FISH) may detect a CD117 expression in gastric carcinoma tumor cells without corresponding IHC results. Miettinen et al. have examined kit-positive angiosarcomas for kit mutations and could not detect any abnormalities in the region typically mutated in GIST [34]. Thus, a positive IHC reaction cannot imply the application of anti kit drug regimes per se. On the other hand, mutations of CD117 genes might be present in the development of gastric carcinomas, and cannot be excluded by our study, as the applied antibodies are only directed against the "wild type" protein formation.

A similar situation was found for bcl-2 expression, which could be demonstrated intra-cytoplasmatically in only one case of a diffuse gastric carcinoma. Thus, the majority of gastric carcinomas (if not all) are immunohistochemically negative for bcl-2. These results are in agreement with the data reported by Wang and his colleagues. The authors reported only 2 bcl-2 positive cases out of 30 gastric carcinomas [47]. Fricke et al. reported a higher percentage of immunohistochemical reactivity (5/24) for bcl-2 in a study performed on 24 cases of diffuse type gastric carcinomas [15]. All of these cases were concurrently positively associated with E-cadherin mutations [15]. But there are increasing reports on Bcl-2 reactivity in gastric cancer with much higher frequency (11, 14). Chen and colleagues reported 80.56% of immunohistochemical reactivity of gastric cancer cells to bcl-2 protein which was irrespective to tumor location, histologic type of cancer, and lymph node metastasis status (11). A report from Brazil shows 45% of IHC reactivity of tumor cells for bcl-2 protein (14). In both studies different types of antibody were used. In Chen study a specific method of assessment was considered for quantitative and qualitative evaluation of IHC reaction for bcl-2 (11). Considering low frequency of reactivity of gastric cancer cells in many reports including that of us, it seems to be rational to perform a large scale and inter-institutional study using specific measurements as electronic measurements to verify the exact rate of bcl-2 reactivity in gastric cancer.

## Conclusion

Summarizing these data and including our own results the expression of CD117 in gastric carcinoma seems to be a very unlikely event and can not practically considered as a potential target of therapy. The expression of bcl-2 in gastric carcinoma seems to be quite rare and currently its clinical significance is unclear. But further large studies needed to perform in order to verify the exact rate of bcl-2 reactivity in gastric carcinoma.
